# Physico-mechanical characteristics of tri-calcium silicate pastes as dentin substitute and interface analysis in class II cavities: effect of CaCl_2_ and SBF solutions

**DOI:** 10.1016/j.heliyon.2019.e01975

**Published:** 2019-06-21

**Authors:** M.M. Radwan, Shaymaa M. Nagi, H.K. Abd El-Hamid

**Affiliations:** a Refractories, Ceramics and Building Materials Department, National Research Centre (NRC), Dokki, Cairo 12622, Egypt; b Restorative and Dental Materials Department, National Research Centre (NRC), Dokki, Cairo 12622, Egypt

**Keywords:** Tri-calcium silicate, Calcium chloride, Simulated body fluid, Hydroxyapatite, Dentin substitute

## Abstract

The influence of using simulated body fluid (SBF) as a curing medium on some characteristics of pure single tri-calcium silicate (C_3_S) bio-cement was investigated. CaCl_2_ salt solution was used as an accelerating liquid for setting and hardening of C_3_S pastes in comparison with distilled water (DW). Solid state reaction was applied to synthesis C_3_S phase at elevated temperature followed by rapid cooling. The results showed that 10 wt.% CaCl_2_ solution was the optimum concentration that showed the lowest setting time (106 min). C_3_S pastes prepared with CaCl_2_ solution have better physical and mechanical properties than those mixed with DW even after curing under SBF solution for the different curing ages. However, SBF solution has an adverse effect on the hydrated compound C-S-H that results in a little decrease in strength and hardness values. The elemental analysis emphasized the presence of apatite layer on the surface of the hardened C_3_S paste. Scanning electron microscopy (SEM) photomicrographs and elemental analysis revealed reliable adaptation of the experimentally prepared C_3_S paste to the tooth structure, in addition to its bioactivity makes it a consistent material to be used as dentin substitute.

## 1 Introduction

Mineral Trioxide Aggregate “MTA” is the first cementitious material that was introduced in dentistry [1]. MTA has wide dental clinical uses as in cases of; perforation repair, root end filling material, direct and indirect pulp capping, formation of apical plug, and management of immature apices [2, 3]. Its properties include dimensional stability, promotion of cementogenesis, good adhesion to tooth structure, bactericidal properties, inductive of hard tissue formation, and bioactivity [4, 5, 6, 7, 8]. Mineral Trioxide Aggregate that is based on calcium silicate is considered as a hydraulic binder. It can set in wet and humid conditions or in some other aqueous solutions, such as saliva, blood or dentinal fluid, forming calcium silicate hydrate gel [9, 10].

MTA products are founded on “Portland Cement” materials, thus they integrally contain mixtures of the main two silicate phases (C_3_S& C_2_S), tricalcium aluminate (C_3_A) and tetracalcium alumino-ferrite (C_4_AF) in addition to small amounts of calcium sulfates (CaSO_4_-gypsum), together with some little amounts of metallic impurities originating from the used raw materials in manufacturing process [1, 2]. Tri-calcium silicate exhibited adequate physico-mechanical properties and can fastly induce the deposition of a bone-like layer of hydroxyapatite on its surface after curing in a simulated body fluid (SBF) [11, 12, 13]. The simulated body fluid (SBF) solution contains different concentrations of inorganic salts similar to those of blood plasma [14].

MTA has some shortcoming as well studies found high solubility after 78 days of storage in water with 24% loss [15, 16]. In addition to its prolonged setting time needs pulp capping to be done with MTA in two clinical visits. A temporary restoration should be placed over MTA to allow it to set before adapting the long-lasting restoration or a fast setting liner is applied to shield MTA during application of permanent restoration. Another draw-backs for MTA is the handling characteristics and its high cost [17, 18].

Recently Active Biosilicate material [Biodentine^TM^ (Septodont, USA)] was introduced in the markets. It was developed to be used as dentin replacement material, restoring furcation perforations, restoration of deep and large coronal, radicular and cervical carious lesions, pulp capping and pulpotomy, repair of root [14]. This product has many advantages compared to MTA. It is a highly pure calcium silicate material formulated by exposing to high temperature. Biodentine is free from any aluminate and other impurities in its composition in contrast to MTA, its aluminate components, make the it fragile. Moreover Biodentine has a short setting time (12min.) compared to MTA that sets within a range of several hours [19, 20]. Decreasing the setting time was done through decreasing the particle size which increases the specific surface, they decreased the overall liquid content in the system and finally calcium chloride was added to the liquid component as an accelerator [19, 20]. One of the most effective set accelerators for the dental cement pastes is calcium chloride. The accelerative power of this salt increases with the increase in its concentration up to certain concentration [18, 21].

Experimentally a highly reactive tri-calcium silicate is prepared in the laboratory at elevated temperatures by solid-state reaction using raw materials unlike those used of Portland cement manufacturing process. Ultra-pure CaO and SiO_2_ are used as raw material to avoid contaminations of any impurities as in case of Portland cement production process [8, 22]. This novel single-phase tri-calcium silicate cement preparation aimed to combine the advantages of both MTA and biodentine with lower cost to be used in dental practice.

The present work aims at studying the influence of using SBF solution as a curing medium on physico-mechanical properties of highly pure and chemically reactive experimental Ca_3_SiO_5_ as a novel single-phase material prepared by solid-state reaction at elevated temperature (1550 °C) to be used as dentin substitute material. Moreover, different concentrations of CaCl_2_ solution which added to tri-calcium silicate (Ca_3_SiO_5_) were used to maintain the minimum setting time for the prepared paste. The material-dentin interface of this novel experimental Ca_3_SiO_5_ was evaluated and compared to commercial Ca_3_SiO_5_ (Biodentine^TM^; Septodont, USA) as dentin substitute material.

## 2 Materials and methods

### 2.1 Material preparation and characterization

C_3_S phase was formulated at the laboratory by firing a homogenized mixture of molar ratio 3:1 CaO:SiO_2_ of chemically pure calcium carbonate (≈99.8%) and (99.6% SiO_2_) respectively, with the addition of 0.5% boric acid at 1000 °C for 2h [23]. The calcined material was crushed, finely ground and remolded using small amounts of CCl_4_. Firing of the composite material at 1550 °C for 2h was repeated several times followed by rapid cooling (at ambient temperature) to ensure the completion of phase formation by investigating the minimum insoluble residue and free lime percentages [12].

The final C_3_S phase was very finely ground in a small quantities (10 gm portions) for 72 h with the aid of zircon ball mill machine (Retsch GmbH PM100, Germany). It has to be mentioned that the synthesized calcium silicate phase may not be affected by the heat liberated during the prolonged milling time as it was prepared at elevated temperature (1550 °C). Thereafter, the structure of the synthesized C_3_S material was emphasized by XRD (Philips X-ray diffractometer PW 1730 with Ni-filtered Cu-Kα X-ray radiation-at 40kV and 30 mA - 2θ: 5°–70° - Δ(2 θ = 0.01°) and TEM (JEOL JEM-2100 (Japan) microscope – at 200kV and 1.402Å resolution).

### 2.2 Setting time

In this study, the preparation of C_3_S pastes was done using two different mixing liquids, i.e. distilled water (DW) and calcium chloride solution. The international standards ISO 9917 1:2007 [24] were used for setting time determination. A W/S ration of 24% was applied for paste preparation using the two mixing solutions [distilled water (DW) and 6%, 8%, 10%, 12%, 14%, 16%, and 18% by weight CaCl_2_]. The setting time was carried out with the aid of a 10mm height mold and modified Vicat apparatus (needle of 1 ± 0.01 mm diameter a mass of 400 ± 5 g) in a cabinet maintained at 37 ± 1 °C temperature and 95% relative humidity. The setting time was checked at 15 min intervals until the needle did not penetrate the cement surface [14].

### 2.3 Compressive strength

A half inch steel molds were used to formulate the C_3_S pastes mixed with both distilled water or 10% CaCl_2_ solution at the same W/S ratio of setting time test. Equal layers of the prepared pastes were poured into the molds then compacted and pressed homogenously along the surface of the mold. Curing after molding was completed in an incubator at 37 °C and 100% relative humidity first for 24h, then the samples was kept at 37 °C under the 2 curing media used in this study which were distilled water (DW) and SBF solution until the desired test time (1, 3, 7and 28 days). The strength values were average of three readings. The strength machine was of the type LLOYD Instrument, Model LR 10K.

The SBF solution used in this investigation was prepared according to Kokubo *et al* [25]; by dissolving reagent grade given in Table 1 in deionized water at pH = 7.4 buffered with Tris-(hydroxyl methyl)-amino methane [(CH_2_OH)_3_CNH_3_] and hydrochloric acid.

**Table 1:** Ion concentration (mM) in the simulated body fluid (SBF) and human plasma Kokubo *et al*[25].

Solution	Ion concentration (mM)							
	Na^+^	K^+^	Mg^2+^	Ca^2+^	Cl^-^	HCO^3-^	HPO_4_^2-^	SO_4_^2-^
SBF	142.0	5.0	1.5	2.5	147.8	4.2	1.0	0.5
Body plasma	142.0	5.0	1.5	2.5	103.0	27.0	1.0	0.5

### 2.4 Water absorption (WA)

C_3_S specimens were formulated by either mixing with (DW) or 10% CaCl_2_ solution with dimensions (15 mm diameter and 2 mm height). Specimens were cured in either DW or SBF solution for (1, 3, 7 & 28 days). Five replicate specimens of the tested materials were made.

The water absorption test was done for all hardened pastes cured at different curing ages by weighting the specimens as dry weight at 100 °C for 24 h and saturated surface dry weight. The water absorption of the hardened specimens was calculated by using the following equation [26]:

*WA* = [(*Wsat*−*Wdry*)/*Wdry*] × 100 %

where.*WA* = Water absorption of hardened specimens, %.*W sat* = Saturated surface dry weight of specimens, g.*W dry* = Dried weight of specimen, g.

### 2.5 Micro-hardness

Micro-hardness testing was performed at two different time intervals (24 h and 28 days). Ten samples of 15 mm diameter and 2 mm thickness were mixed using 10% CaCl_2_ solution and cured for one day at 37 ± 1 °C in an incubator. The samples were subjected to micro-hardness test (24 h) using Vickers micro-hardness instrument (Nexsus 4503, Innova Test, Netherlands, Europe). Three randomized indentations were made with a 100-gram load for10 s. Before indentations and for accuracy, the samples were arbitrarily rotated. The micro-hardness values were calculated in accordance with: Hardness-Course Vickers/Brinell/Rockwell copy right IBS 2012 version 10.4.4. After that the tested specimens were divided into two groups referring to the immersion media; either SBF or DW for 28 days. After immersion in both SBF and DW for 28 days, specimens were dried in a desiccator and finer grits of silicon carbide paper, from 180-grit to 1200-grit was used to ground the surface of all discs then polished with 3-micron polycrystalline diamond paste [14]. The determination of micro-hardness for these specimens was done again as was discussed above.

### 2.6 pH measurements

The 10% calcium chloride solution was used to prepare discs of C_3_S paste of 15 mm diameter and 2 mm height. The prepared C_3_S were cured in an incubator at 37 ± 1 °C for 24 hours and then immersed in 20 ml of SBF solution in a sealed plastic containers. The pH values of each immersion solution after 1, 3, 7 and 28 days were measured with the aid of solid-state pH sensor connected to a pH meter (Medika Scientific Jenway bench top pH meter, England). pH was determined by insertion of the electrode in the curing solution at ambient temperature (24 °C). Five replicate specimens were tested and the pH measurement was recorded three times. Standard buffer solutions (pH 4.01, 7.01 and 10.00) were used for pH meter calibration before measurements [14, 27, 28].

### 2.7 FTIR spectroscopy

The FTIR spectral analysis was done on some hardened pastes using FTIR spectrometer of model Jasco-4600.

### 2.8 Material-dentin interface analysis using SEM and EDX analysis

The Ethics Committee of the National Research Centre, Cairo, Egypt approved the Sound and caries-free premolars recorded for extraction for orthodontic reason that were used in the investigation.

In each tooth, a mesial and distal box-shaped class II cavity was prepared with a cylindrical diamond bur in a high-speed hand-piece using copious water-cooling. No bevels were prepared, and all margins were placed in enamel. The depth of the occlusal cavity was around 2 mm, width of the cavity was 2 mm, and the length of the axial wall was 6 mm. The prepared cavities of each tooth were arbitrarily assigned to one of the two experimental groups; experimental Tri-calcium silicate cement mixed with 10% calcium chloride solution, and a commercial Tri-calcium silicate cement [Biodentine^TM^ (Septodont, USA)]. All restorations were made by one operator. After one-month immersion in SBF, the premolars were sectioned in buccal-lingual direction.

The interface of each restoration to dentin was evaluated at 1500x magnification using scanning electron microscopy (SEM)[Model Quanta 250 FEG (Field Emission Gun)] coupled with energy dispersive x-ray spectroscopy (EDX) (FEI company, Netherlands) [29].

### 2.9 Statistical analysis

For each group in each test, the mean and standard deviation values were calculated. Kolmogorov-Smirnov and Shapiro-Wilk tests were used to explore the data for normality as it showed parametric (normal) distribution. Repeated measure ANOVA was used to compare between more than two groups in related samples. Paired wise sample t-test was used to compare between two groups in related samples. One-way ANOVA followed by Tukey post hoc test was used to compare between more than two groups in non-related samples. Independent sample t-test was used to compare between two groups in non-related samples. The significance level was set at p ≤ 0.05. Statistical analysis was performed with IBM® SPSS® Statistics Version 20 for Windows.

## 3 Results

### 3.1 Characterization of the prepared tri-calcium silicate (C_3_S)

**Fig. 1:**
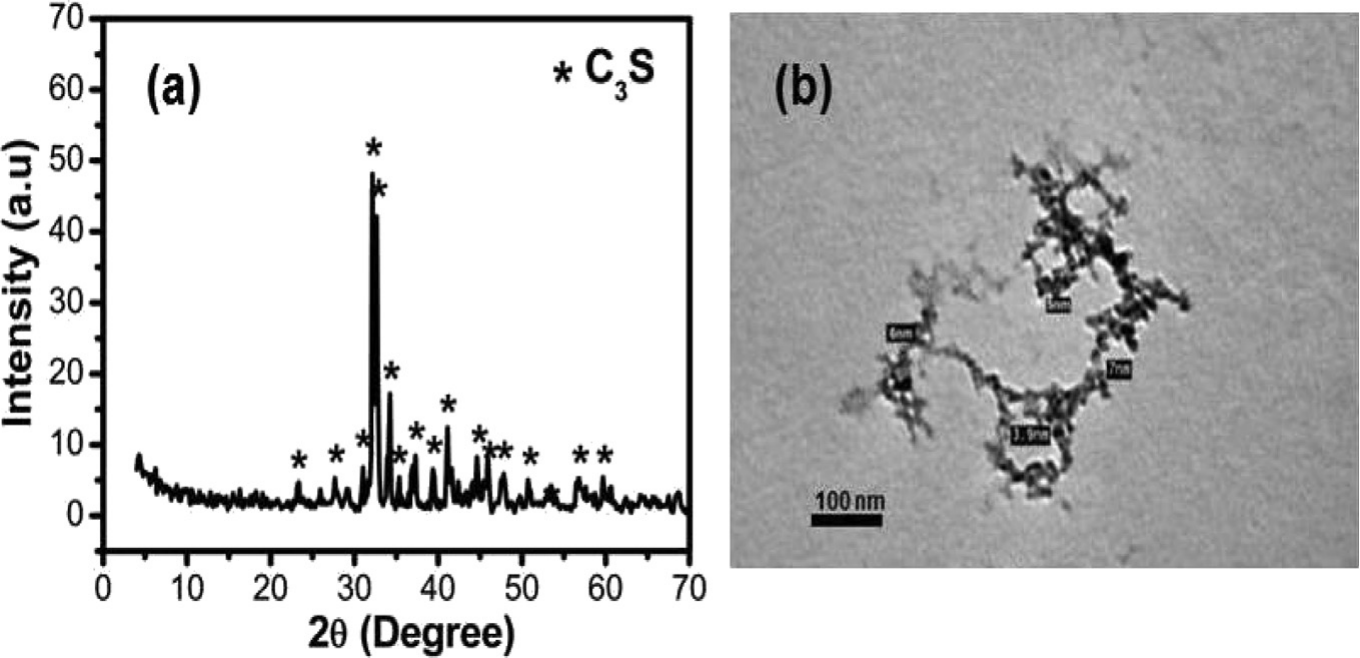
(a) XRD patterns and (b) TEM of the prepared C_3_S powder.

Fig. 1a&b shown the XRD patterns and TEM of C_3_S phase respectively. The prepared tri-calcium silicates had been emphasized by XRD analysis, according to the standard JCPDS No.: 55–0738 (Fig. 1a) [30]. From TEM analysis (Fig. 1b), we deduced that the particle size of the synthesized C_3_S powder is ranged from 4-7 nm.

### 3.2 Setting time

**Fig. 2:**
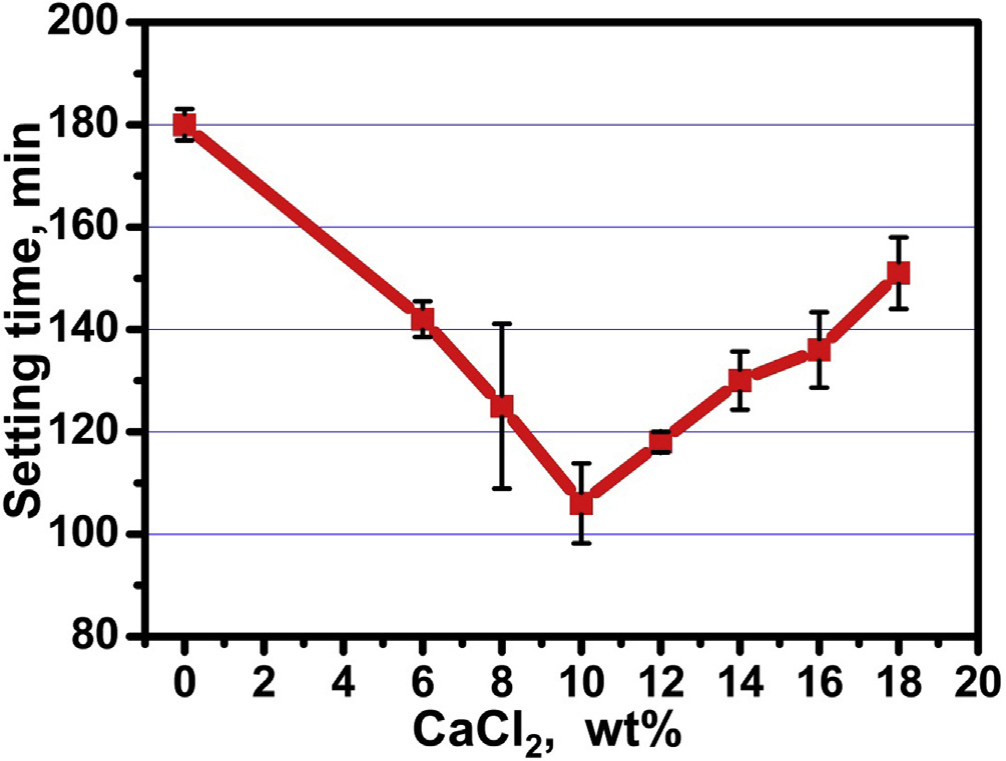
Setting-time of the prepared calcium silicate phase mixed with different concentrations of calcium chloride salt solution (6%, 8%, 10%, 12%, 14%, 16%, and 18% by wt. CaCl_2_).

Table 2 and Fig. 2. represents the influence of using calcium chloride solutions as an accelerator for setting process of the prepared calcium silicate phase.

**Table 2:** Mean, and standard deviation (SD) of setting time in different groups.

Variables	Setting time (min)	
	Mean	SD
C_3_S + distilled water	180.67	3.06
C_3_S + 6% CaCl_2_	141.67	3.51
C_3_S + 8% CaCl_2_	125.00	16.09
C_3_S + 10% CaCl_2_	106.00	7.81
C_3_S + 12% CaCl_2_	118.00	2.00
C_3_S + 14% CaCl_2_	129.67	5.69
C_3_S + 16% CaCl_2_	134.33	7.37
C_3_S + 18% CaCl_2_	151.00	7.00
*p-value*	<0.001*	

*; significant (p < 0.05) ns; non-significant (p > 0.05).

A statistically significant difference was found between the tested groups at *p* < 0.001. C_3_S specimens mixed with DW showed the highest significant mean value (180.67 ± 3.06 min). On the other hand; C_3_S specimens mixed with 10% CaCl_2_ showed the least significant mean value (106 ± 7.81 min), compared to (C_3_S/14% CaCl_2_), (C_3_S/16% CaCl_2_) and (C_3_S/18% CaCl_2_) at (*p* = 0.030), (p = 0.007) and (p < 0.001) respectively.

### 3.3 Compressive strength

**Fig. 3:**
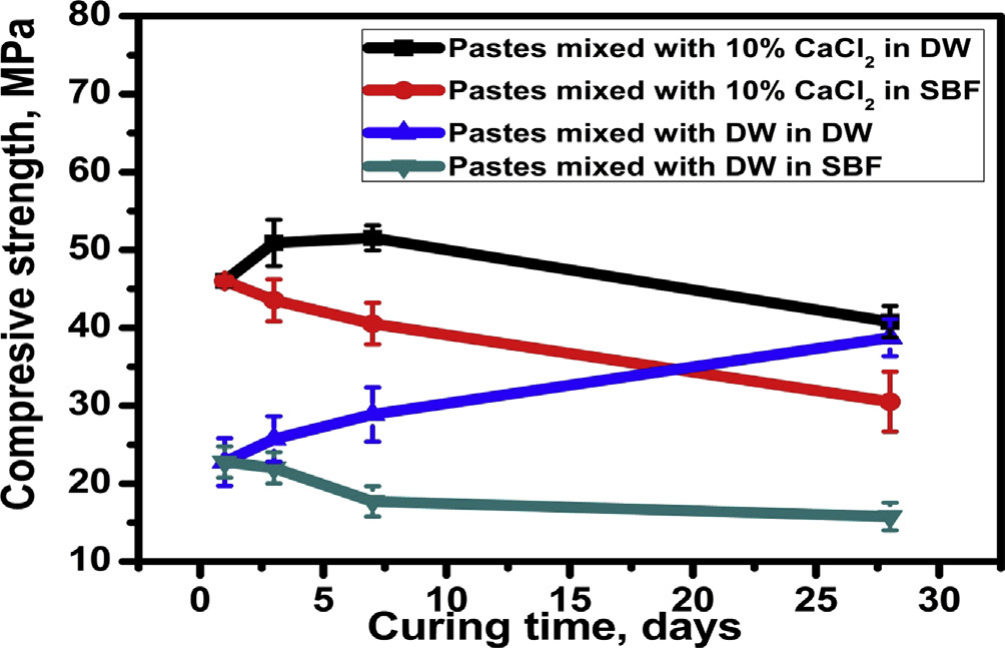
Compressive strength values of pastes mixed by distilled water or calcium chloride solution and cured in both DW and SBF solution.

The compressive strength mean ± SD values of C_3_S specimens either mixed with DW or 10% CaCl_2_ solution and cured either in DW and SBF solution are given in Table 3 and Fig. 3. All tested groups showed a statistically significant difference in mean compressive strength (MPa) between (day 1), (day 3), (day 7) and (day 28) at *p* < 0.001*.

**Table 3:** Mean, standard deviation (SD) of compressive strength in different groups.

Variables	Compression (MPa)								
	C_3_S/CaCl_2_/DW		C_3_S/CaCl_2_/SBF		C_3_S/DW/DW		C_3_S/DW/ SBF		p-value
	Mean	SD	Mean	SD	Mean	SD	Mean	SD	
Day 1	45.37^b^	0.74	45.23^bc^	0.60	23.87^b^	3.07	23.40^a^	1.99	<0.001*
Day 3	50.90^ab^	2.98	43.40^b^	2.69	26.37^b^	2.90	22.07^ad^	2.00	<0.001*
Day 7	52.03^a^	1.62	41.63^ca^	2.67	29.90^b^	3.48	18.17^bd^	1.95	<0.001*
Day28	42.43^b^	2.02	32.77^a^	3.85	38.63^a^	2.38	16.20^cd^	1.78	<0.001*
*p-value*	0.019*		0.013*		0.034*		0.012*		

Different letter within each column indicates significant difference, * = Significant, NS = Non-significant.

Regarding comparing the effect of the mixing solutions; results revealed a statistically significant difference between C_3_S specimens mixed with (DW) and (CaCl_2_) at (*p* < 0.001). Specimens mixed with 10 % CaCl_2_ showed the highest compressive strength mean value (44.22 ± 6.05MPa) while specimens mixed with DW showed the least compressive strength mean value (24.83 ± 7.06 MPa). On the other hand; on comparing the effect of the curing solutions, results revealed a statistically significant difference between the curing solutions (DW and SBF) at *p* = 0.013, where specimens cured in DW showed the highest compressive strength mean value (38.69 ± 10.65 MPa) while specimens cured in SBF showed the least compressive strength mean value (30.36 ± 11.54 MPa).

### 3.4 Water absorption (WA)

**Fig. 4:**
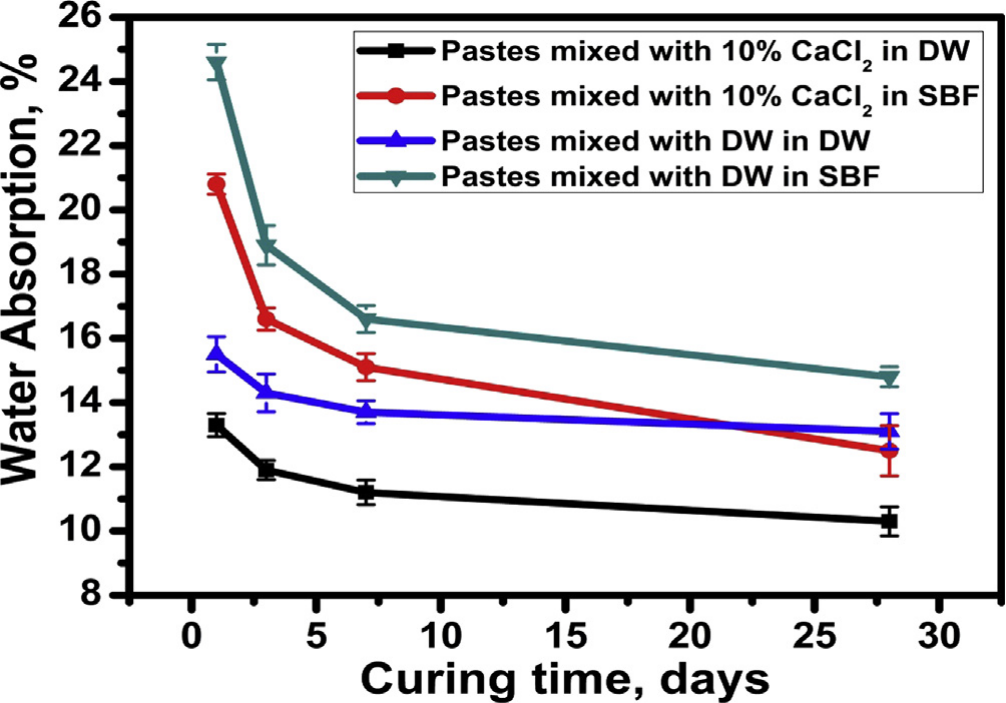
Water absorption (WA) values of C_3_S pastes prepared with both distilled water (D.W) and 10% CaCl_2_ solution cured in DW and SBF.

The water absorption (WA) mean ± SD values of C_3_S pastes prepared with both distilled water (DW) and 10% CaCl_2_ solution cured either in DW or SBF solution are represented in Table 4 and Fig. 4. In all groups a statistically significant difference was found between (day 1) (day 3) (day 7) and (day 28) at *p* < 0.001*.

**Table 4:** Mean, and standard deviation (SD) of water absorption in different groups.

Variables	Water absorption (%)									
	C_3_S/CaCl_2_/DW		C_3_S/CaCl_2_/SBF		C_3_S/DW/DW		C_3_S/DW/ SBF			p-value
	Mean	SD	Mean	SD	Mean	SD		Mean	SD	
Day 1	13.3^a^	0.36	20.83^a^	0.31	15.53^a^	0.55		24.67a	0.55	<0.001*
Day 3	11.8^b^	0.30	16.67^b^	0.35	14.37^ab^	0.59		18.90b	0.61	<0.001*
Day 7	11.33^b^	0.38	15.03^c^	0.42	13.80^b^	0.36		16.63db	0.42	<0.001*
Day28	10.33^c^	0.45	12.50^d^	0.78	13.53^b^	0.55		14.87c	0.31	<0.001*
*p-value*	0.006*		<0.001*		0.014*			<0.001*		

Different letter within each column indicates significant difference, * = Significant, NS = Non-significant.

Regarding comparing the effect of the mixing solutions; results revealed a statistically significant difference between C_3_S specimens mixed with (DW) and (CaCl_2_) at (*p* = 0.013). Specimens mixed with DW showed the highest water absorption mean value (16.54 ± 3.58) while specimens mixed with 10 % CaCl_2_ showed the least water absorption mean value (13.98 ± 3.31). On the other hand; on comparing the effect of the curing solutions on the mean water absorption, results revealed a statistically significant difference between the curing solutions (DW and SBF), SBF curing solution showed the highest water absorption mean value (17.51 ± 3.70) while DW showed the least water absorption mean value (13 ± 1.68).

### 3.5 Micro-hardness

**Fig. 5:**
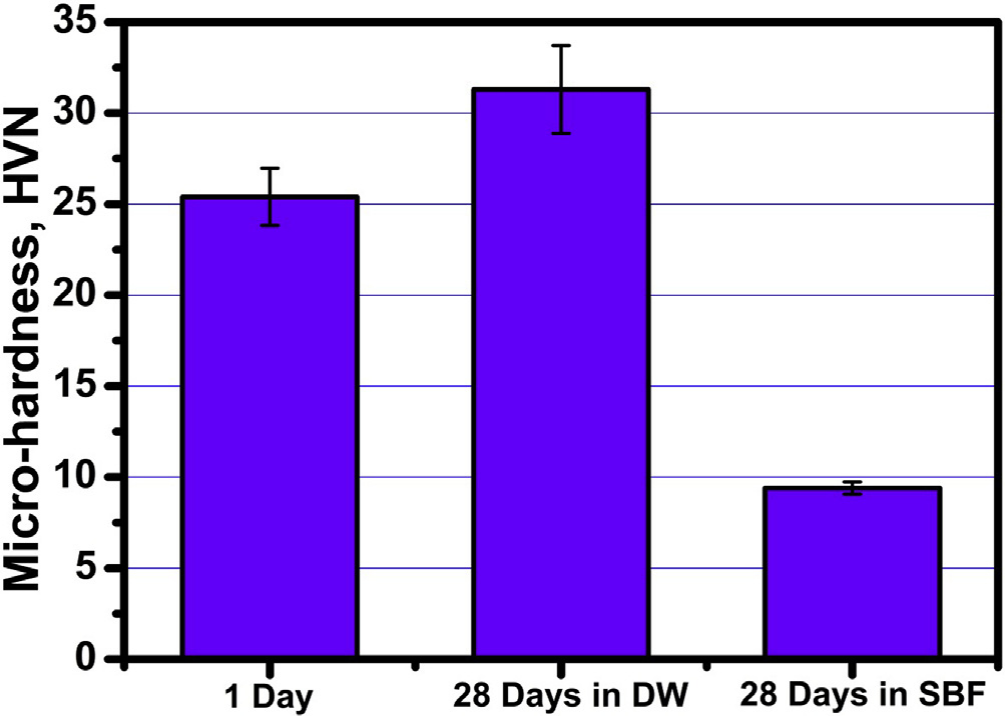
Micro-hardness values of specimens prepared with 10% CaCl_2_ liquid cured for one day and 28 days in both SBF solution and DW.

The micro-hardness mean ± SD values of specimens prepared with 10% CaCl_2_ liquid cured for 1 and 28 days in both SBF solution and DW are shown in Table 5 and Fig. 5. For specimens cured in DW for 28 days; a statistically significant increase in the mean micro-hardness values (VHN) was shown in comparison to (day 1) specimens. On the other hand; specimens cured in SBF showed statistically significant decrease in the mean micro-hardness values (VHN) in comparison to (day 1) specimens.

**Table 5:** Mean, standard deviation (SD) of micro-hardness in different groups.

Variables	Micro-hardness (HVN)					
	DW			SBF		p-value
	Mean	SD		Mean	SD	
Day 1	25.42	1.57		25.42	1.57	1ns
Day 28	31.60	2.42		9.24	0.34	<0.001*
p-value	0.001*		<0.001*			

*; significant (p < 0.05) ns; non-significant (p > 0.05).

A statistically significant difference was found between (day 28 DW) and (day 28 SBF) at (*p* = 0.024). The highest micro-hardness mean value (VHN) was found in (day 28 DW) while the lowest mean value was found in (day 28 SBF).

### 3.6 pH measurement

**Fig. 6:**
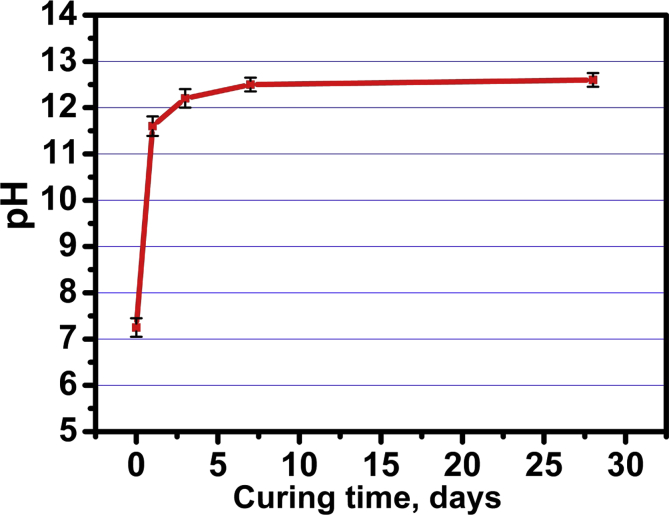
pH variations of the SBF solution due to hydration process of C_3_S pastes prepared with 10% calcium chloride solution.

Table 6 and Fig. 6. shows the pH mean ± SD values of the SBF solution due to hydration process of C_3_S pastes prepared with 10% calcium chloride solution. A statistically significant difference was found between (day 1) (day 3) (day 7) and (day 28) where (*p* = 0.002). The highest mean value was found in day 28 (12.53 ± 0.15) followed by (day 7) and (day 3) with no statistically significant difference between them between at (p = 0.076), while the lowest mean value was found in (day1) (7.27 ± 0.21).

**Table 6:** Mean, and standard deviation (SD) of pH in SBF groups.

Variables	PH	
	Mean	SD
Day 1	7.27	0.21
Day 3	11.60	0.20
Day 7	12.23	0.15
Day 28	12.53	0.15
*p-value*	0.002*	

*; significant (p < 0.05) ns; non-significant (p > 0.05).

### 3.7 Infrared spectroscopy

**Fig. 7:**
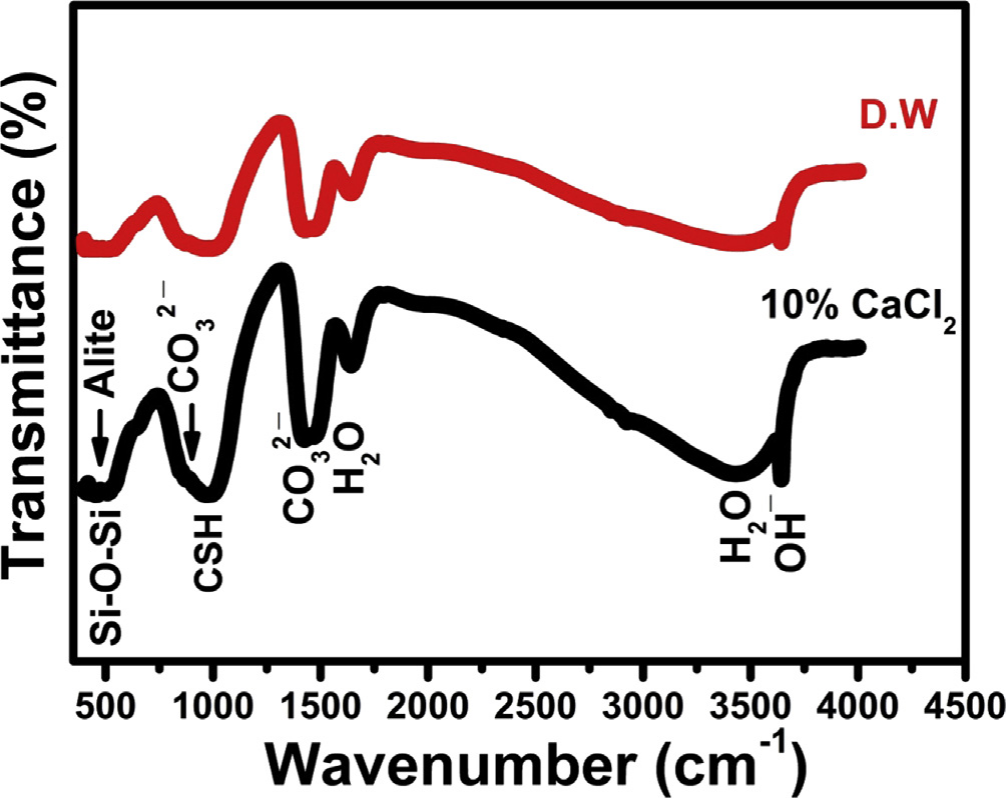
Infrared bands of pastes mixed with D.W and CaCl_2_ cured for one day.

The FTIR spectral analysis was used in this study to emphasize the role of CaCl_2_ solution in accelerating the hydration reactions and shortening the setting time of calcium silicate single phase. The infrared bands of pastes mixed with distilled water and 10% calcium chloride solution after 24 h curing period are given in Fig. 7. The IR bands given in Fig. 7 indicate that the main Si-O-Si stretching vibration bands at 490 cm^−1^ corresponding to the anhydrous C_3_S phase (Alite) has slightly lower intensity for pastes mixed with CaCl_2_ solution than those prepared with distilled water (DW).

On the other hand, a slight increase of the C-S-H (calcium silicate hydrate) IR bands intensities located at 445, 815 and 950 cm^−1^ was detected. The IR bands intensities of all hydrated phases at 950 cm^−1^ and incorporated ν_2_H_2_O and OH^−^ at 1630&3365 cm^−1^ and 2500&3700 cm^−1^, respectively, corresponding to the main hydrated C-S-H gel of C_3_S cement were also enhanced by using the calcium chloride solution as mixing liquid. The intensity of the characteristic IR bands of Aragonite (CO_3_^2-^ antisymmetric stretching) at 1460-1490 cm^−1^ that are found as a result of the possible carbonation process of the hydrated phases by the action of atmospheric carbon dioxide are slightly greater for pastes prepared with the CaCl_2_ solution due to the accelerated rate of early hydration reactions and formation of more hydrated compounds and liberation of more free Ca(OH)_2_ in reaction medium.

**Fig. 8:**
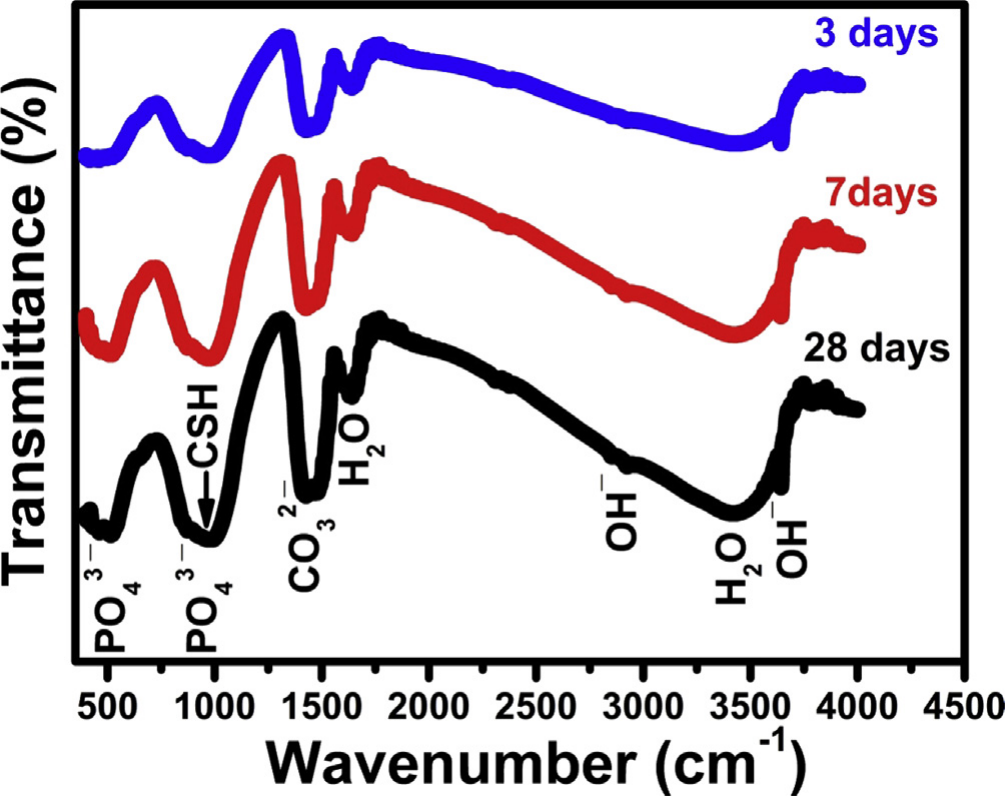
Infrared bands for pastes mixed with DW and cured in SBF Solution for 3,7,28 days.

**Fig. 9:**
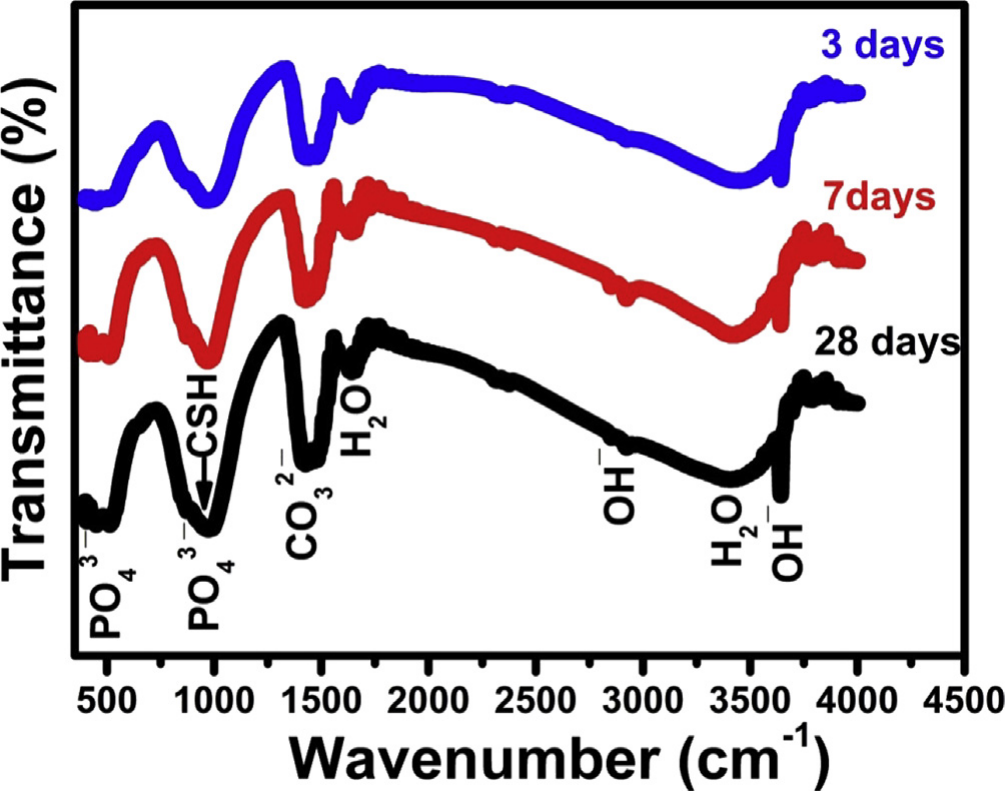
Infrared bands for pastes mixed with CaCl_2_ solution and cured in SBF solution for 3,7,28 days.

Figs. 8 & 9 show the IR spectra of pastes prepared with both DW and 10% CaCl_2_ solution cured for 3, 7, and 28 days. The IR bands showed the presence of phosphate group IR band at 1030 cm^−1^ (antisymmetric stretching) due to the formation of hydroxyapatite bio material together with those of OH^−^ stretching band at 490, 3648, 2500 and 3700 cm^−1^ corresponding to the molecular water for all hydrated phases. The IR data in the two figures emphasizes that all the above mentioned IR bands characteristic for hydrated compounds are increased with the curing period and also, they are slightly larger for samples prepared with the accelerating agent (10 % CaCl_2_ solution) as mixing liquid. The same trend of increasing IR band intensity could be detected for all hydrated sample as the calcium chloride solution did enhances the rate of hydration reactions of C_3_S phase especially at early hydration age.

### 3.8 Results for the material-dentin interface analysis using SEM- EDX

**Fig. 10:**
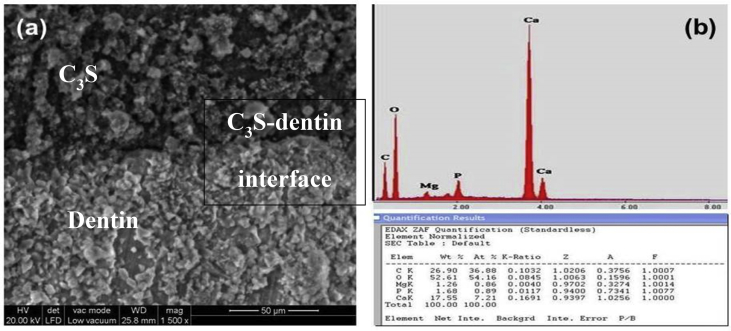
(a) SEM photomicrograph and (b) EDX analysis for experimental tri-calcium silicate-dentin interface.

**Fig. 11:**
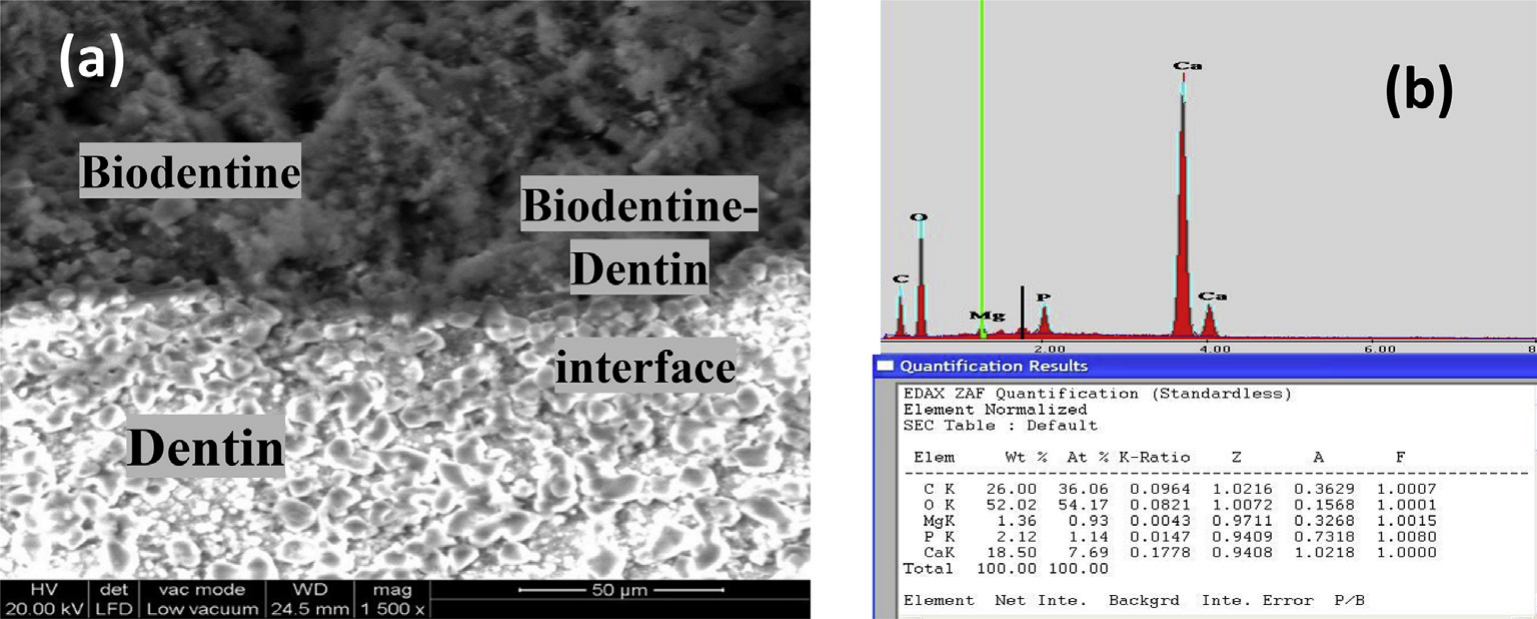
(a) SEM photomicrograph and (b) EDX analysis for Biodentine-dentin interface.

Fig. 10a &11a show the SEM photomicrographs of the comparable material-dentin interface of the experimental tri-calcium silicate cement and the commercial biodentine dentin substitute material respectively. Both showed surface deposits of crystals at the cement-dentin interface with no gap formation. Figs. 10b & 11b show the EDX analysis of experimental tri-calcium silicate-dentin interface and Biodentine-dentin interface. Results of EDX analysis indicates the presence of calcium and phosphate ions at both cement-dentin interface.

## 4 Discussion

Ultra-pure tri-calcium silicate (C_3_S) is considered the main silicate phase of mineral trioxide material (MTA). Preparation of this single phase in a highly reactive form was done by firing the molar ratio of the reactant oxides (CaO& SiO_2_) at 1550 °C and rapid cooling of the resulting phase to room temperature. The hydration process of the formulated silicate phase started immediately by mixing the dry phase powder with distilled water this resulted in formation of the main hydrated compound, that is, calcium silicate hydrate (C-S-H) and liberation of free lime (Ca(OH)_2_) as by-product in accordance to Eq. (1) [21, 23].

(1) 3CaO.SiO_2_ + 5.3 H_2_O→1.7CaO.SiO_2_.4H_2_O + 1.3Ca(OH)_2_

During setting and hardening of C_3_S paste, it solidifies in a rigid structure of excellent mechanical properties. Normally, some special admixtures may be used in certain concentration with distilled water in order to accelerate the setting and hardening processes. Calcium chloride (CaCl_2_.2H_2_O) is well known in medical and dental applications as an accelerating agent for both setting and hardening of silicate bio-materials. Both Ca^2+^and Cl^¯^ anions may contribute to the acceleration of the hydration reactions of calcium silicate materials. Flocculation of hydrophilic colloids influenced by charges repulsion of ions and water molecules keeping them apart. The same effect may be transferred to the growing C-S-H particles [14, 15].

Due to the relatively long setting time (180 min) of C_3_S pastes prepared with distilled water (DW), calcium chloride solutions of concentrations ranging between 6-18 wt.% were applied in this investigation as mixing liquids (Table 2 and Fig. 2) in order to shortening the setting times for this type of biomaterials to be useful for dental applications. Referring to the above mention discussion concerning the role of cations and anions (Ca^2+^and Cl^¯^) in accelerating setting process, it was found that by increasing the concentration of CaCl_2_ solution from 6 to 10% the setting time decreased from 180 to 106 min and by increasing the concentration from 10% to 18% the setting time increased to 151min.The increase of the setting time by increasing the concentration of calcium chloride solution may be attributed to the common ion effect that may cause repulsion of the increasing amount of free cations and anions in the hydration medium [23].

In this investigation, curing of C_3_S pastes mixed with DW and the optimum calcium chloride solution (10wt.% CaCl_2_) was done under simulated body fluid (SBF) of chemical composition given in Table 1. The influence of SBF solution on hydration process, hardening process and some physico-mechanical properties was investigated. Table 6 and Fig. 6. indicates the large increase of pH values of pastes mixed with calcium chloride solution after 7 days curing age as a result of the acceleration effect of CaCl_2_ on the hydration reaction due to liberation of an increasing amounts of free lime (Ca(OH)_2_) in SBF solution (eq. 1). It was stated that this increasing amount of free lime have superior antimicrobial activity that make this type of bio-cements have an excellent bioactivity for both dental applications and bone repair [6, 30, 31]. The IR spectra given in Fig. 7 emphasizes the acceleration effect of calcium chloride for the setting process and hydration reactions during the early 24 h of hydration more than using distilled water (DW) as mixing liquid this is clear from the IR bands enhancement of the hydrated C-S-H compound at 950 cm^−1^ and those characteristic of the incorporated ν_2_ H_2_O at 1630 & 3365 cm^−1^ and 2500 & 3700 cm^−1^ for pastes prepared with CaCl_2_ solution [23, 31].

As was mentioned above, curing and hydration of C_3_S pastes mixed with DW and CaCl_2_ solution after storage in incubator at 37 °C was completed to 28 days curing age in SBF solution. SBF solution was applied in this study to determine the apatite layer formation on the surface of hardened calcium silicate paste cured in SBF which was detected by EDX analysis (Fig. 10b) [13, 14, 20, 21]. The presence of various cations and anions in SBF solution should affect the hydration characteristics and physico-mechanical properties of the formulated C_3_S phase. Table 1 represents the ion concentrations (mM) of all cations and anions namely, Na^+^, K^+^, Mg^+^, Ca^2+^, Cl^−^, HCO^3-^, HPO_4_^2-^ and SO_4_^2-^ in SBF solution. Some of these cations when found in low concentration can accelerate the hydration process of C_3_S cement in the order, Ca^2+^˃ K^+^˃ Na^+^, while Mg^+^, HCO^3-^, HPO_4_^2-^ and SO_4_^2-^ may have an aggressive attack and deleterious effect on the main hydrated phases of C_3_S material [23]. In this case there are two opposing effects that may affect the hardened pastes, one can enhance the phase hydration reactions and the other may adversely affect the physical and mechanical properties of final rigid structure [21].

The strength values (Table 3 and Fig. 3) of hardened pastes mixed with 10% CaCl_2_ solution were higher at almost all curing ages than those prepared with DW that is expected due the role of calcium chloride in enhancement the early hydration reactions of calcium silicate phase in addition to the presence of the free cations and anions in SBF solution that have also effective behavior in accelerating the hardening process at later curing periods. The normal compressive strength increased from one to 28 curing ages for pastes prepared with DW cured in DW was not that case for those mixed with the calcium chloride solution which might be attributed to the encapsulation of the hydrated compounds around the anhydrous particles due the accelerated hydration reaction rate at the first few hours after mixing. This is followed by the carbonation process that is happened by the reaction of dissolved carbon dioxide (CO_2_) in the pore system with Ca^2+^ and OH^¯^ free ions obtained from the dissolution of the liberated free lime (Ca(OH)_2_) and by lowering the Ca/Si ratio of the C-S-H to form CaCO_3_. The formed calcium carbonate will precipitate in the micro-pores resulting in a deleterious effect on the physical and mechanical properties due to the hair cracks formed in the final hardened structure [21, 23].

As was mentioned above, the presence of some aggressive ions in SBF solution showed an opposite adverse effect on physical and mechanical properties (Tables 3, 4, and 5, Figs. 3, 4, and 5) that is mainly responsible for the strength loss of all hardened pastes either prepared with DW or CaCl_2_ solution. C_3_S pastes are normally attacked by low concentrated Na_2_SO_4_solution resulting in the formation of CaSO_4_ (gypsum) due to the presence of Ca^2+^ in the pore solution from the dissolved Ca(OH)_2_. The formed gypsum precipitated in the pore system resulting in a hair cracks and strength loss. Also, the presence of acidic groups such as HCO^3-^ and HPO_4_^2-^ in hydration medium are highly aggressive for the main C-S-H hydrated compound which will dissolve it. The presence of MgCl_2_ may also attack the C_3_S paste due to the formation of basic salts (Mg(OH)_2_) by the reaction with dissolved Ca(OH)_2_, the free Mg^2+^ cations will replace Ca^2+^ in C-S-H magnesium silicate hydrate (Mg-S-H) which has very poor mechanical properties [21]. This aggressive attack mentioned above could be discussed in terms of water absorption and hardness results given in Tables 4 and 5 & Figs. 4 and 5, as they were adversely affect by curing under SBF solution but they are still reasonable for dental and medical applications. The IR spectra (Figs. 8 and 9) can emphasize the findings of the physical and mechanical data. The IR band at 1030 cm^−1^ (antisymmetric stretching) of hydroxyapatite layer formed on the surface of hardened pastes cured in SBF solution was detected in IR spectra of Fig. 9. Also, the role of using calcium chloride to accelerate the setting and hardening processes is clear from the increase of intensities of IR bands of C-S-H at 445, 815 and 950 cm^−1^and those of OH^−^ stretching band at 490, 3648, 2500 and 3700 cm^−1^ corresponding to the molecular water for all hydrated phases. As an evidence for the carbonation of the hydrated phases, the characteristic IR bands of Aragonite (CO_3_^2−^antisymmetric stretching) at 1460-1490 cm^−1^ was detected in all IR spectra.

### 4.1 Discussion for SEM and EDX analysis

The SEM photomicrographs (Figs.10a & 11a) of the present study showed comparable material-dentin interface for both the experimental tri-calcium silicate cement and the commercial biodentine dentin substitute material. This could be justified due to various reasons, as both materials are based on tri-calcium silicate cements which has an ability to form hydroxyapatite crystals on the surface of the cement on hydration and dissolving slowly in SBF solution [32, 33], which was confirmed by EDX analysis that indicates the presence of calcium and phosphate ions (Figs.10b&11b). Several studies support our finding regarding the bioactivity of biodentine material [19, 20, 34].

Rapid ionic exchange of Na^+^ or K^+^ with H^+^ or H_3_O^+^from the SBF solution occurred when a bioactive silica-based (tri-calcium silicate) material was immersed in it. This interchange caused a silica hydrogel (Si(OH)_4_) layer created on the interface of materials solution with increasing the pH value of the solution. When the pH value increase, portion of the silica hydrogel was softened, that lead to breakage of Si-O-Si bonds and the creation of silanols (Si-OH) groups, which in turn form dense and re-polymerized SiO_2_-rich layer on the surface. Passage of Ca^2+^ and PO_4_^2-^ groups to the superficial layer across the SiO_2_-rich layer formed heterogeneous nucleation of the initial calcium phosphate [35, 36, 37]. This process continues as the cement ages and helps to close the gap between the cement-tooth interface. It micromechanically bonds to the tooth without any prior surface treatment of the tooth surface. Moreover; on account of the high alkaline nature of the ca-silicates, it causes caustic erosion of the dentin and penetrates into the dentinal tubules and adheres to dentin. Although there is initial contraction of cement during hydration there is a secondary expansion of the cement, explaining the sealing ability [29]. The advantage of using a calcium silicate-based material for dentin replacement is leaching of calcium hydroxide from the set cement [38, 39]. Its biocompatibility adds to the other properties of the cement and makes it a reliable material to be used as dentin substitute material in deep cavities, in close proximity to the underlying pulp tissue.

## 5 Conclusions

- The pure single C_3_S phase (4–7 nm) was formulated by solid state reaction at 1550 °C and rapid cooling with good chemical reactivity.
- Using of 10 wt.% CaCl_2_ solution as mixing liquid did accelerate the setting and hardening processes and shortening the long setting time of C_3_S pastes to its lower value (106 min).
- C_3_S pastes mixed with 10 % CaCl_2_ solution and cured under D.W showed better physical and mechanical properties even those cured for different curing periods under SBF solution.
- Using SBF solution as curing medium adversely affect the mechanical strength and hardness of the hardened pastes due to the aggressive attack of some free cations and anions (Mg^+^, HCO^3-^, HPO_4_^2-^ and SO_4_^2-^) present in the hydration medium, while, their values still high enough for this bio-materials to be used in dental applications.
- The reliable adaptation of the experimentally prepared C_3_S paste to the tooth structure, in addition to its bioactivity makes it a consistent material to be used as dentin substitute material.

## Declarations

### Author contribution statement

M. M. Radwan, Shaymaa M. Nagi: Conceived and designed the experiments; Performed the experiments; Analyzed and interpreted the data; Contributed reagents, materials, Analysis tools or data; Wrote the paper.

H.K. Abd El-Hamid: Performed the experiments; Analyzed and interpreted the data; Contributed reagents, materials, Analysis tools or data.

### Funding statement

This work was supported by the National Research Centre (NRC), Egypt (Grant No. AR 111403).

### Competing interest statement

The authors declare no conflict of interest.

### Additional information

No additional information is available for this paper.
